# Leaving Against Medical Advice: Current Problems and Plausible Solutions

**DOI:** 10.7759/cureus.64230

**Published:** 2024-07-10

**Authors:** Shamma S Alhajeri, Ibrahim A Atfah, Ali M Bin Yahya, Salama M Al Neyadi, Maryam E Al Nuaimi, Fatema S Al Ameri, Nasser Ahmed, Ismail M Al Ramahi, Kenneth C Dittrich, Hasan Qayyum

**Affiliations:** 1 Emergency Department, Sheikh Khalifa Medical City, Abu Dhabi, ARE; 2 Emergency Department, Sheikh Khalifa Medical CIty, Abu Dhabi, ARE

**Keywords:** clinical audit, united arab emirates (uae), emergency departments, discharge planning, leave against medical advice

## Abstract

Leave against medical advice (LAMA) is defined as ‘a decision to leave the hospital before the treating physician recommends discharge’, and is associated with higher rates of readmission, longer subsequent hospitalization, and worse health outcomes. In addition to this, they also contribute to poor healthcare resource utilization. We conducted a single-center audit to establish patient demographics and contributing factors of patients leaving against medical advice from our emergency department (ED). We benchmarked our data against locally available clinical policy guidelines. We interrogated our electronic health record system (known as Salamtak^®^), which is a Cerner-based platform (Cerner Corporation, Kansas City, MO 64138) for patients who signed LAMA from ED from 2018 to 2023. We selected a convenience pilot sample of 120 subjects. Based on a literature review, we identified patient demographics (age, gender, nationality, socioeconomic status, marital status, religion), possible contributing factors (time of attendance, insurance status, length of ED stay), and patient outcomes (reattendances within 1 week and mortality) to evaluate. Based on locally available guidance, we formulated six criteria to audit with a standard set at 100% for each. A team of emergency medicine residents collected data that was anonymized on an Excel spreadsheet (*Microsoft Excel, *Microsoft Corporation. (2018). Basic descriptive statistics were used to collate results. About 93 patients (77.5%) were 16 years and above, and 27 patients (22.5%) were below 16 years. There was a slight preponderance of males (64 patients, 53.3%) than females (56 patients, 46.6%). The majority of LAMA cases presented in the evening and night (97 patients, 80.8%). About 57 (47.5%) patients had an ED length of stay of 3 hours or more. The average ED length of stay for these patients was 3.4 hours. About 73 patients (60.3%) were insured. Out of 120 patients, only 12 (10%) had a mental capacity assessment documented. The commonest reason for signing LAMA was a social reason in 45 (37.5%) cases. In the remaining cases, the causes were a combination of family, financial, waiting, or other/undocumented reasons). When faced with a decision to LAMA, the involvement of a Public Relationship Officer (PRO) was only documented to be consulted in seven (5.8%) cases. About 14 cases were re-attended within 1 week (11.6%) and no mortalities were reported in any of the reattendances. LAMA is a not-so-rare phenomenon often occurring in EDs, and often a cause of trepidation for healthcare workers. Treating this as an aberrant behavior on the part of the patient, or laying the responsibility for this action on the healthcare provider is primitive, counter-productive, and not patient-centric. Familiarity with local guidelines around this contentious area is essential. Revised nomenclature like ‘premature discharge’ may be less stigmatizing for the patient. Where possible, a harm reduction approach should be used and frontline healthcare workers must be prepared with an escalation plan. In the United Arab Emirates, familiarity with Wadeema’s Law as a child protection measure is essential.

## Introduction

Leave against medical advice (LAMA), also referred to as discharge against medical advice (DAMA), has been defined as ‘a decision to leave the hospital before the treating physician recommends discharge’ [1]. The prevalence of LAMA has been reported as 0.07% to 20% in emergency departments (ED) worldwide [2]. LAMA has also been reported to have higher rates of readmission, longer subsequent hospitalization, and more importantly worse patient outcomes [3]. In addition to this, they are also associated with poor healthcare resource utilization, with 50% higher costs reported when readmitted after leaving against medical advice [4].

Our healthcare network in the Abu Dhabi Emirate of the United Arab Emirates (UAE), known as ‘SEHA’, the Abu Dhabi Health Services Company, published a ‘General Discharge Policy’ in March 2023 [5]. We used this and an additional locally sourced clinical policy guideline from Tawam Hospital, Al Ain [6] to benchmark our practices at Sheikh Khalifa Medical City Emergency Department. We also wanted to raise awareness about the UAE’s ‘Wadeema Law’ which the general discharge policy refers to. The law, in line with the Convention of the Rights of the Child, stresses that all children must be provided with appropriate living standards, access to health services, education, equal opportunities, and access to essential services and facilities, without any kind of discrimination. The law protects children against all forms of negligence, exploitation, physical and psychological abuses, and covers all aspects of children’s rights in the UAE, with the necessary instruments to ensure its effective implementation.

There are many reasons why a patient may choose to LAMA like long waiting times, financial constraints, family obligations, disagreement/dissatisfaction with the treatment plan, or a perceived improvement in their condition [5]. The American College of Emergency Physicians reports patients who LAMA to be at high risk for malpractice litigation with a reported prevalence of one lawsuit per 300 against medical advice cases [7-9].

We conducted a single-center audit to identify patient demographics and contributing factors of patients leaving against medical advice from our ED.

## Materials and methods

We interrogated our electronic health record system for patients who signed LAMA from ED from 2018 to 2023. We selected a convenience pilot sample of 120 subjects. Based on a literature review, we identified patient demographics (age, gender, nationality, socioeconomic status, marital status, religion), possible contributing factors (time of day of attendance, insurance status, length of ED stay, insurance status), and patient outcomes (reattendances within 1 week and mortality) to evaluate. As this was part of a service evaluation, formal ethics approval was not required.

Based on locally available guidance [5,6], we formulated six criteria to audit. We proposed a standard of 100% for each criterion. These are listed in Table 1.

**Table 1:** Our audit criteria each with a standard set at 100%

Criteria		Standard
Criterion 1	All patients opting to leave against medical advice will have a LAMA form signed by the patient or their legal guardian.	100%
Criterion 2	All patients opting to leave against medical advice will have a Salamtak LAMA powernote completed.	100%
Criterion 3	All adult patients opting to leave against medical advice signing LAMA will have a formal assessment of capacity done.	100%
Criterion 4	All patients opting to leave against medical advice will have a reason for LAMA documented (e.g., social, financial/insurance, treatment delay, personal)	100%
Criterion 5	All patients opting to leave against medical advice will have an alternative plan offered to them (e.g., review by another physician)	100%
Criterion 6	All patients opting to leave against medical advice will have a follow-up plan discussed.	100%

Results were collected on an Excel spreadsheet (Microsoft Corporation (2018) Microsoft Excel). Basic descriptive statistics were used to collate the results.

## Results

We used a traffic light system to highlight areas of good practice and those requiring improvement. A score of 0-79% was considered non-compliant, 80-89% partially compliant, and 90-100% fully compliant. Based on this system, we were fully compliant with Criterion 1 (96%), partially compliant with Criterion 4 (87%), and non-compliant with Criteria 2, 3, 5, and 6 (19%).

Patient demographic characteristics of the 120 patients are summarized in Table 2.

**Table 2:** Patient demographics and possible contributing factors for ‘leave against medical advice’

Age (N)	Less than 16 = 27		16 and above = 93
Gender (N)	Male = 64		Female = 56
Marital status (N)	Married = 59		Non-married = 61
Nationality (N)	National = 50		Non-national = 70
Length of stay (N)	Less than 3 hours = 57		More than 3 hours = 63
Insurance (N)	Present = 73		Absent = 47
Mental health history (N)	Present = 6		Absent = 114
Time of attendance (N)	1400-2200 hours	2200-0600 hours	0600-1400 hours
	Evening = 56	Night = 41	Day = 23

About 93 patients (77.5%) were 16 years and above, and 27 patients (22.5%) were below 16 years. There was a slight preponderance of males (64 patients, 53.3%) than females (56 patients, 46.6%). The majority of LAMA cases presented in the evening and night (97 patients; 80.8%). A total of 57 (47.5%) patients had an ED length of stay of 3 hours or more. The average ED length of stay for these patients was 3.4 hours. A total of 73 patients (60.3%) were insured.

Out of the 120 patients, only 12 (10%) had a mental capacity assessment documented, all of whom were above the age of 16 years. The commonest reason for signing LAMA was a social reason in 45 (37.5%) cases. In the remaining cases, the causes were a combination of family, financial, waiting, or other/undocumented reasons). When faced with a decision to LAMA, the involvement of a Public Relationship Officer (PRO) was only documented to be consulted in seven (5.8%) cases. About 14 cases were re-attended within 1 week (11.6%) and no mortalities were reported in any of the reattendances. Audit results are collated in Table 2.

## Discussion

The decision to ‘LAMA’ or ‘DAMA’ is common across healthcare networks and associated ED. This poses a challenge to the emergency physician who on one hand must respect the patient’s right to LAMA, but then weigh it against their competent ability to make this decision, and consider the effects of incomplete management [10]. Our study of 120 patients found the decision to LAMA was commoner in the male population. Young age, male gender, low socioeconomic status, HIV infection, substance misuse, insurance level, and concurrent medical and psychiatric co-morbidities are patient-specific risk factors for against medical advice discharges [11,12].

It is well-described that LAMA is associated with negative patient outcomes. Such patients have a higher risk of readmission within 30 days, up to 40% higher [3]. The readmission is often for the same diagnosis but with higher clinical needs, requiring a higher level of care [13]. There is also a higher risk of 30-day mortality, by approximately 10% [14]. Patient dissatisfaction with care is commonly associated with the decision to LAMA. This may be due to issues with individual healthcare care staff, communication breakdown, and delayed waiting times to see physicians [15-19]. It’s also important to note patients leaving against medical advice are less likely to return for follow-up and do not receive planned discharge care. These patients are less likely to have access to a primary care physician [20,21]. It’s reported that nearly 20% of patients who LAMA feel reluctant to return due to chances of perceived disdain on return and potential conflict with healthcare workers [19]. From a revenue perspective, DAMA is expensive as the cost for the primary hospital visit and readmission is more than 50% higher than the primary hospitalization alone [13]. Such patients also have a longer length of stay on their subsequent reattendances than those who have planned discharges [22].

Previous studies have shed light on the complex etiology of LAMA. Patients may leave if the patient or even their caregivers have a disagreement about their clinical management. Another reason cited is navigating the complex maze of referrals and consults so the patient can eventually be consulted by the right physician. This results in at least a prolonged transit time in ED for such patients, and also a lack of clarity in the patient’s management plan when so many teams are consulted [23]. Failure to manage expectations, especially in patients with sickle cell disease where pain control is an active complaint, can lead to a breakdown in the rapport between patients and physicians [24,25]. Poor customer service, lack of respect, and poor quality of care, as perceived by the patient to be due to the cultural background and the healthcare team are also known to contribute [26]. Often it is noted that nursing staff are left with completing paperwork and documentation for patients leaving against medical advice. As voices for the patient, they should be center stage in drafting policies and interventions to address the root causes of LAMA [23]. Past medical history is important to establish a correlation with LAMA. History of substance misuse, including alcohol and IV drug use, heart failure, asthma, and HIV infection are also associated with reports of LAMA [27,28]. One study reported the incidence of DAMA in patients affected by substance abuse to be 20-54%. Psychiatric illness, especially in young single men has been associated with a higher risk of LAMA [29]. Patients with HIV are also known to be associated with a higher risk of LAMA, from 10-30% of cases. This has been attributed to non-compliance with treatment, lack of social support, and difficulty accessing regular medical care [28,30,31]. Asthmatic patients, most being young males, with low-income backgrounds, and uninsured are also associated with higher rates of LAMA. An interesting but unfortunate observation was patients with asthma who left against medical advice were twice as more likely to require critical care admission and intubation [17]. In our study, approximately a third of patients who opted to LAMA were uninsured. In some studies, lack of insurance or the need to self-pay was a common cause for patients leaving against medical advice [32,33].

Tackling LAMA is a complex task due to the intertwining of patient, provider, and healthcare system factors, most of which are either poorly understood or remain unknown [11]. A new thought process focuses on a shared patient-system responsibility model to bring system-based learning to the foyer in order to bridge existing quality gaps [11]. Currently, it is common practice for healthcare staff to believe they are absolved of their legal and ethical responsibilities by designating a discharge as against medical advice [28]. This is often regarded as not patient-centric, stigmatizes the patient, and moves away from a shared decision-making model. New nomenclature would suggest replacing leave against medical discharge with a more neutral term, i.e. ‘premature discharge’ [11]. One of the spectrums is apportioning blame on the patient. On the other end, it is also unwise to put responsibility solely on the healthcare staff. This will create what is termed as a ‘second victim’ effect by blaming physicians and other providers for an event they have little control over [11]. It is suggested, and perhaps this might appear aspirational, that the responsibility of managing the expectations of patients, addressing their valid concerns, sharing clinical decisions, and arranging alternate follow-up for patients with such premature discharges be shared by the healthcare network [11].

One of the key areas our study focused on was the appropriate documentation of the process of leaving against medical advice. This should include a formal assessment of capacity. Local hospital or healthcare network-wide procedures must be adhered to. It is recommended the LAMA documentation includes capacity assessment, the discussion between the physician and the patient, an explanation of risks and benefits, reasons for leaving against medical advice, alternatives offered, advice they can return to the ED anytime, notification to their primary physician, and evidence of a harm reduction approach [5].

Tackling ‘LAMA’ requires a parallel approach where the patient’s interests are kept first and harm reduction strategies are instituted. It is always helpful to seek out why the patient wants to leave without being judgmental. Try and address patient concerns and needs. A parallel step would be to institute harm reduction strategies. This could involve consulting a different physician, arranging a consult, and supporting patients with their treatment goals, providing the patient some safety netting by providing prescriptions, follow-up, and informing about the facility to return [5], this is summarized in Table 3.

**Table 3:** Tackling ‘LAMA’ requires a parallel approach where the patient’s interests are kept first and harm reduction strategies are instituted LAMA: leave against medical advice

Keep the patient first	Harm reduction strategies
Explore reasons why leave against medical advice is being sought.	Support the patient in their treatment goals.
Avoid being judgmental on reasons why the patient is leaving.	Can the patient be seen by another physician or another healthcare facility?
Does the patient have the capacity to make this decision?	Can follow-up be arranged in the clinic?
Seek senior’s help.	Provide the patient with a summary of their medical reports.
Involve the Public Relationship Officer.	Explain they can return to the ED anytime.
Seek assistance from the legal team if in doubt.	Document in detail.

In the UAE, Wadeema’s Law in line with the Convention of the Rights of the Child stresses that all children must be provided with appropriate living standards, access to health services, education, equal opportunities, and access to essential services and facilities, without any kind of discrimination. When patients up to the age of 18 years, and/or their carers opt to LAMA, the same procedure of a harm reduction approach should be followed. The services of a PRO can also be sought to assist in counseling about treatment choices. However, in addition to this, our policy advises should the condition be deemed life-threatening for the child and the carer cannot be convinced, we should involve security and police in the best interests of the child as a matter of child protection [5].

Our study was a relatively small sample-sized retrospective cohort study with a convenience sample collected which would make the results difficult to generalize to a wider population. It should also be noted at the outset that the results reflected in our audit are influenced by the quality of documentation recorded and reviewed as part of this project. Nevertheless, we believe this has sparked an interesting discussion within our emergency medicine community in the UAE, and raised awareness about Wadeema’s Law and around the challenges of LAMA, along with ways to mitigate the risk around it from a patient, physician, and system perspective.

## Conclusions

LAMA is a not-so-rare phenomenon often occurring in ED, and often a cause of trepidation for healthcare workers. Treating this as an aberrant behavior on the part of the patient, or laying the responsibility for this action on the healthcare provider is primitive, counter-productive, and not patient-centric. Familiarity with local guidelines around this contentious area is essential. Revised nomenclature like ‘premature discharge’ may be less stigmatizing for the patient. A model where the responsibility of LAMA is shared between the patient and the healthcare system is the need of the hour. Where possible, a harm reduction approach should be used. An escalation plan should further advice and guidance needed should be readily available, for example, the hospital legal team or a defense union.
